# Pulmonary embolism after SARS-CoV-2 vaccination

**DOI:** 10.1016/j.jvacx.2024.100571

**Published:** 2024-10-15

**Authors:** Björn Zethelius, Sofia Attelind, Gabriel Westman, Rickard Ljung, Anders Sundström

**Affiliations:** a The Swedish Medical Products Agency, Uppsala, Sweden; b Department of Public Health/Geriatrics, Uppsala University, Uppsala, Sweden; c Department of Medical Sciences, Clinical Pharmacogenomics, Uppsala University, Uppsala, Sweden; d Department of Medical Sciences, Infectious Diseases, Uppsala University, Uppsala, Sweden; e Institute of Environmental Medicine, Karolinska Institute, Stockholm, Sweden; f Department of Pharmacy, Uppsala University, Uppsala, Sweden

**Keywords:** COVID-19 vaccines, Vaccine safety, Primary vaccinations, Booster vaccinations, Pulmonary embolism, Public health, Regulatory science

## Abstract

**Background:**

During the COVID-19 vaccination campaign in Sweden, pulmonary embolism (PE) was a frequently reported suspected serious adverse drug reaction. The aim was to estimate risk of PE following vaccination for COVID-19 in the Swedish population aged 18 to 84 years.

**Methods:**

Population-based cohort study using the CoVacSafe-SE established platform including national registers. PE-case definition: Individuals discharged from inpatient-care or visiting specialized outpatient-care with a main diagnosis of PE occurring between 27-Dec-2020 and 31-Dec-2022 without simultaneous diagnosis of COVID-19 infection. Time-to-event analysis was performed using multi-variable Cox’ proportional hazard’s models. Hazard Ratios (HR) adjusted for age, sex and co-morbidities were modelled.

The vaccines were BNT162b2/Comirnaty®, mRNA1273/Spikevax® and ChAdOx1 nCoV-19/Vaxzevria® without regard to variants. Doses number one to five were studied.

**Results:**

Eighty percent of the study-population (≈6.1 million people) received at least two doses of COVID-19 vaccine. A total of 12,456 cases of PE were identified. Twenty-eight days after vaccinations we observed 99 cases after 701,455 1st doses of ChAdOx1 nCoV-19, HR_adj_, 1.29 (95%-CI, 1.05–1.59). Corresponding for BNT162b2 was 361 cases after 4,708,284 1st doses of BNT162b2 HR_adj_ of 1.19 (95%-CI, 1.06–1.34) driven by age group 65–84; HR _adj_, 1.24 (95%-CI, 1.07–1.44). No increased risks were observed for mRNA1273.

**Conclusion:**

In this nation-wide study, no strong associations were found between COVID-19 vaccinations and pulmonary embolism. Small increases in relative risk for the earliest doses of vaccines may be associated with prioritizing the frailest groups of people in the vaccination campaign, thus selection bias or unmeasured residual confounding is possible.

## Introduction

Symptoms of SARS CoV-2 infection are highly variable, ranging from none to life-threatening critical illness. COVID-19 have been linked to risk of pulmonary embolism (PE), a serious medical emergency where blood clots block the pulmonary arteries, leading to impaired blood flow to the lungs and possible death [1], [2], [3], [4]. Such a risk is particularly pronounced in the acute phase of COVID-19 and then diminishing with time but still elevated after six months [1].

Intense and rapid vaccine development was launched early in the pandemic [5] and vaccines were rapidly implemented in national vaccination programs world-wide after accelerated approval processes. The following four vaccines were used in Sweden: Two mRNA vaccines (BNT162b2 and mRNA-1273) and approximately 6 times more individuals were vaccinated with BNT162b2 than with mRNA-1273 because of higher availability of the former; a viral vector vaccine (ChAdOx1 nCoV-19) was mainly used for those age 65 years and above for primary vaccinations before restrictions in use were launched and a protein subunit vaccine (NVX-CoV2373) was used to a very limited extent.

Sweden implemented a phased vaccine distribution plan in January 2021, initially prioritising vaccination of those at highest risk of COVID-19 complications and those at high risk of exposure and transmission whereafter all residents in Sweden 16 years and older have been eligible for vaccination. Studies on effectiveness in the Nordic countries have shown high protection against COVID-19 related hospitalizations among adolescents after primary vaccinations [6], among adults aged 50 years and above after bi-valent booster [7] and increased protection against severe omicron-related COVID-19 outcomes among all adults aged 18 years and above after heterologous booster schedule [8].

A large population exposure achieved in a very short time requires systematic monitoring of safety. Thus, the Swedish Medical Products Agency (SMPA) launched the COvid-19 VACcination register SAFEty study in Sweden (CoVacSafe-SE) [9].

During the COVID-19 vaccinations in Sweden in 2021 to 2022, PE was a frequently spontaneously reported suspected acute serious adverse drug reaction (ADR) to the SMPA. A study from Singapore [10] with 42 days follow-up after vaccinations with BNT162b2 or mRNA-1273, revealed no increased risk for PE. Two studies in people aged 65 years and above from the US did not observe increased PE-risk with 28 days of follow-up [11] or inconsistent evidence for PE after COVID-19 mRNA primary vaccinations or monovalent boosters with 28 days of follow-up [12]. Further, a study from France up to 14 days after BNT162b2 vaccinations revealed no increased risk for PE [13] nor did a study from Israel on BNT162b2 up to 42 days after primary vaccinations [14]. There is a lack of large studies on PE-risk after primary- as well as booster-vaccinations with longer follow-up.

The aim of the present study was to estimate the risk of the hard endpoint PE during six months following primary and booster vaccinations, up to dose 5, for the COVID-19 vaccines used in the entire Swedish population aged 18 to 84 years, using national registers of high validity and completeness.

## Material and methods

Population based cohort study performed in Sweden from 27 December 2020 to 31 December 2022. The cohort included all individuals aged 18–84 years old, registered in Sweden since 31 December 2015, and still alive on start of follow-up, 27 December 2020. Nationwide healthcare register data were used, i.e., the CoVacSafe-SE platform for epidemiological surveillance set up by the SMPA [9].

The following registries were used: National vaccination register (NVR) and SmiNet (Positive PCR test for SARS-CoV-2); Public Health Agency, Sweden. Swedish National Prescribed Drug Register (PDR), Swedish National Patient Register (NPR), Swedish National Cancer Register (SCR) and Swedish National Cause of Death Register (CDR); National Board of Health and Welfare. Total Population Register (TPR); Statistics Sweden. Linking individual level exposure data of SARS-CoV-2 vaccinations with the other health data registers was made using the unique personal identity number assigned to all Swedish residents.

### Exposure and risk windows

The risk of an outcome event of PE was analysed separately after each dose number (1, 2, 3, 4, and 5) for respective: 7-, 14-, 28-, 60-, 90-, and 180-days risk windows after respective vaccination-day for the latest dose, considered day 1.

Four vaccines, granted marketing authorisation (MA) in the European Union [15], were used in Sweden:

– Comirnaty®; BNT162b2 mRNA vaccine by Pfizer-BioNTech (MA, December 2020).
– Spikevax®; mRNA-1273 mRNA vaccine by Moderna Biotech Spain (MA, January 2021).
– Vaxzevria®; ChAdOx1 nCoV-19 viral vector vaccine by Oxford-AstraZeneca (MA, January 2021).
– Nuvaxovid®; NVX-CoV2373 SARS-CoV-2 subunit spike-protein by NOVAVAX CZ (MA, December 2021).

### Study outcome

We defined incident outcome event as the date of a discharge from inpatient care (including death from PE) or the date for a specialized outpatient care visit using NPR with a main diagnosis of pulmonary embolism (PE), i.e. the International Statistical Classification of Diseases and Related Health Problems (ICD)-code I26, between 27 December 2020 and 31 December 2022. Cases had to be free of any record of PE in the past five years. The Swedish NPR have a good accuracy for the PE diagnosis that may be reliably used in register research [16].

### Statistics

Individuals were followed up until first occurring diagnosis of PE, censored at a positive COVID-19 test, the sixth COVID-19 vaccination dose, emigration, death from other causes or end of follow-up.

For the primary vaccination (doses 1 and 2), the whole population was followed from the start of the vaccination campaign, December 27th, 2020. For the risk associated with the third dose, the population receiving the second dose formed the study population, and the follow-up started at the date of the administration of the second dose. For the risk associated with the fourth and fifth dose, the study-population was those who received the third dose, and follow-up started on the date of administration of the third dose.

Time-to-event analysis using multi-variable Cox’ proportional hazard’s models were performed using Statistical Analysis System; SAS Enterprise Guide 8.2. Hazard ratios (HR) with 95 % confidence intervals (CIs) are presented, adjusted for sex and age and for a full model. Full model adjustments were made for the following: Sex, age, marital status, country of birth, main and secondary diagnoses in in- or out-patient care in the five years preceding follow-up (since 2015) for possible risk factors for the outcome: Deep vein thrombosis, an important predisposing condition for PE; any cancer/malignancies; Type 1- and 2-Diabetes and Cardiovascular Diseases; Obesity; Chronic Obstructive Pulmonary Disease (COPD) and nicotine replacement therapy; Inflammatory Bowel Disease (IBD); Chronic autoimmune disease; Severe renal failure, chronic renal failure, stage 5; Severe liver failure; using immunosuppressives for a previous transplant; Thrombophilia; Inpatient hospital care for any diagnosis other than PE as a marker of frailty; use of pharmaceutical drugs, i.e. numbers per individual of any drug as a marker of frailty; anti-coagulants; antidiabetic medicines, anti-obesity medicines; medicines for chronic autoimmune diseases; combined oral contraceptives and hormone replacement therapy (Variable definitions, using ICD-10 codes for diseases and ATC-codes for pharmaceuticals used, are listed in Appendix Table A4).

Covariates had to be registered as main or secondary diagnosis in either in- or out-patient care during the past five years. For diagnoses registered in out-patient specialist care two different visits with the same diagnosis code were required.

## Results

The total population, 12–84 years old consisted of 7,513,618 individuals (49.7 % women) observed for 13,087,019 person years at risk (PYAR). Eighty percent of the study-population, 6,072,228 individuals received at least two doses of COVID-19 vaccine, i.e. the primary vaccinations scheme and 12,456 cases of PE were identified during the study period. Three percent of PE cases died within 30 days (n = 374) from PE-diagnosis. Mean duration between dose one and two was 48 days and between dose two and dose three was 196 days. Among the mRNA-vaccines, BNT162b2 was the dominant (85 %) product used, due to higher availability, for primary vaccinations and for booster doses. The ChAdOx1 nCoV-19 was used for primary vaccinations mainly for those aged 65 years and above with approximately 70 % of the ChAdOx1 nCoV-19 doses administered to this group. Use of the NVX-CoV2373 vaccine was very limited.

Table 1 show total population characteristics on contemporary diseases and pharmaceuticals used in panel A and age and follow-up time on cases of PE in panel B.

**Table 1:** **A-B.** Population characteristics for the total population (A) under study and the pulmonary emboli outcome population (B).

Panel A	All	Women	Men
Individuals (N,%)	7,513,618	3,734,899 (49.7 %)	3,778,719 (50.3 %)
Follow-up time (person-years)	13,087,019	6,448,116	6,638,903
Age (years; mean, SD)	49.3 (18.2)	49.8 (18.3)	48.9 (18.1)
Age (Q1; median; Q3)	34; 49; 64	34; 50; 65	33; 49; 64
*Marital status*			
Married	3,055,967 (40.7 %)	1,529,882 (20.4 %)	1,526,085 (20.3 %)
Unmarried	3,252,080 (43.3 %)	1,478,715 (19.7 %)	1,773,365 (23.6 %)
Divorced	949,515 (12.6 %)	534,363 (7.1 %)	415,152 (5.5 %)
Widow, −er	256,056 (3.4 %)	191,939 (2.6 %)	64,117 (0.9 %)
*Country of birth*			
Sweden	6,071,525 (80.8 %)	3,007,546 (40.0 %)	3,063,979 (40.8 %)
Europe excl Sweden	640,337 (8.5 %)	333,265 (4.4 %)	307,072 (4.1 %)
Rest of the world	801,756 (10.7 %)	394,088 (5.2 %)	407,668 (5.4 %)
*Number of drugs dispensed^1^*			
Minimum	0	0	0
Q1/Q2 border	0	1	0
Median	2	3	1
Q3/Q4 border	5	6	4
Maximum	74	74	71
Combined oral contraceptives^2^	261,251 (3.5 %)	261,232 (7.0 %)	19 (0.0 %)
Hormone-therapy^3^	487,933 (6.5 %)	486,846 (13.0 %)	1,087 (0.0 %)
Testosterone	29,622 (0.4 %)	3,458 (0.1 %)	26,164 (0.7 %)
Various hormones^4^	34,111 (0.5 %)	33,557 (0.9 %)	554 (0.0 %)
Any hormone above, composite	792,502 (10.5 %)	765,106 (20.5 %)	27,396 (0.7 %)
Ciklosporin or tacrolimus (transplanted)5	10,666 (0.1 %)	4,272 (0.1 %)	6,394 (0.2 %)
Previous DVT^6^	46,509 (0.6 %)	21,711 (0.6 %)	24,798 (0.7 %)
Antithrombotic treatment^7^	290,423 (3.9 %)	114,427 (3.1 %)	175,996 (4.7 %)
Nicotine replacement therapy^8^	91,178 (1.2 %)	53,158 (1.4 %)	38,020 (1.0 %)
Diagnosis COPD^9^	104,403 (1.4 %)	56,746 (1.5 %)	47,657 (1.3 %)
COPD & nicotine, composite variable	182,018 (2.4 %)	101,743 (2.7 %)	80,275 (2.1 %)
Diabetes (diagnosis)^10^	273,530 (3.6 %)	110,351 (3.0 %)	163,179 (4.3 %)
Diabetes (treatment)^11^	462,486 (6.2 %)	190,192 (5.1 %)	272,294 (7.2 %)
Cardiovascular diagnoses^12^	410,974 (5.5 %)	153,163 (4.1 %)	257,811 (6.8 %)
Diabetes & CV, composite variable	785,250 (10.5 %)	315,079 (8.4 %)	470,171 (12.4 %)
Obesity (diagnosis)^13^	216,650 (2.9 %)	150,470 (4.0 %)	66,180 (1.8 %)
Obesity (surgery)^14^	25,792 (0.3 %)	19,724 (0.5 %)	6,068 (0.2 %)
Obesity (drugs)^15^	19,901 (0.3 %)	14,703 (0.4 %)	5,198 (0.1 %)
Obesity, composite variable	231,231 (3.1 %)	161,130 (4.3 %)	70,101 (1.9 %)
Autoimmune disease^16^	185,831 (2.5 %)	122,357 (3.3 %)	63,474 (1.7 %)
Treatment for autoimmune diseases^17^	58,198 (0.8 %)	32,283 (0.9 %)	25,915 (0.7 %)
Autoimmune, composite variable	31,194 (0.4 %)	19,920 (0.5 %)	11,274 (0.3 %)
Inflammatory bowel disease^18^	87,041 (1.2 %)	43,122 (1.2 %)	43,919 (1.2 %)
Cancer^19^	226,785 (3.0 %)	107,279 (2.9 %)	119,506 (3.2 %)
Severe renal failure^20^	7,941 (0.1 %)	2,819 (0.1 %)	5,122 (0.1 %)
Severe hepatic disease^21^	7,270 (0.1 %)	4,076 (0.1 %)	3,194 (0.1 %)
Thrombophilia^22^	8,708 (0.1 %)	6,197 (0.2 %)	2,511 (0.1 %)
Any hospital care during follow-up	1,008,536 (13.4 %)	571,271 (15.3 %)	437,265 (11.6 %)
**Panel B**			
Cases of pulmonary embolism (N, %)	12,456	6,041 (48.5 %)	6,415 (51.5 %)
Follow-up time (person-years)	11,758	5,737 (48.8 %)	6,021 (51.2 %)
Age (mean, SD)	66.8 (13.7)	67.5 (14.4)	66.2 (13.1)
Age (Q1; median; Q3)	60; 71; 77	62; 72; 78	59; 69; 76

*Notes:* 1–22: see Appendix Table A4 for definitions.

**Fig. 1:**
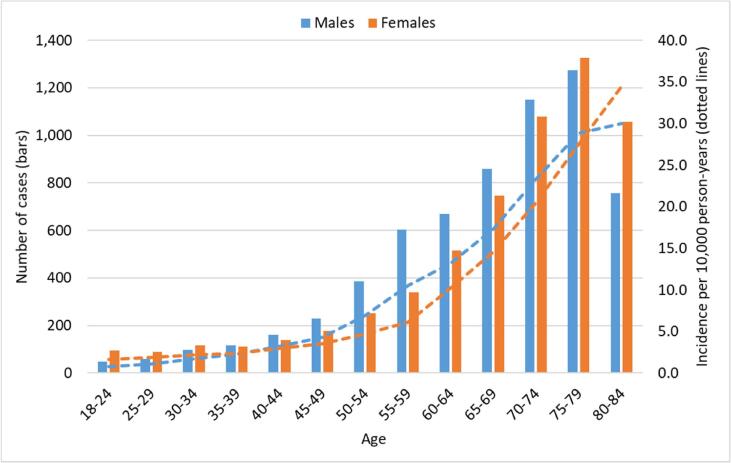
Number of pulmonary emboli cases (bars) during the study period per 5-year intervals for women and men from 18 to 84 years of age and corresponding incidence per 10,000 person-years (dotted lines).

Fig. 1 present number of PE-cases during the study period per 5-year age intervals for women and men. Slightly higher frequencies were observed in women in childbearing ages up to age 35 and from age 75 and above. Between 35 and 74 years, men showed increased frequencies.

**Fig. 2:**
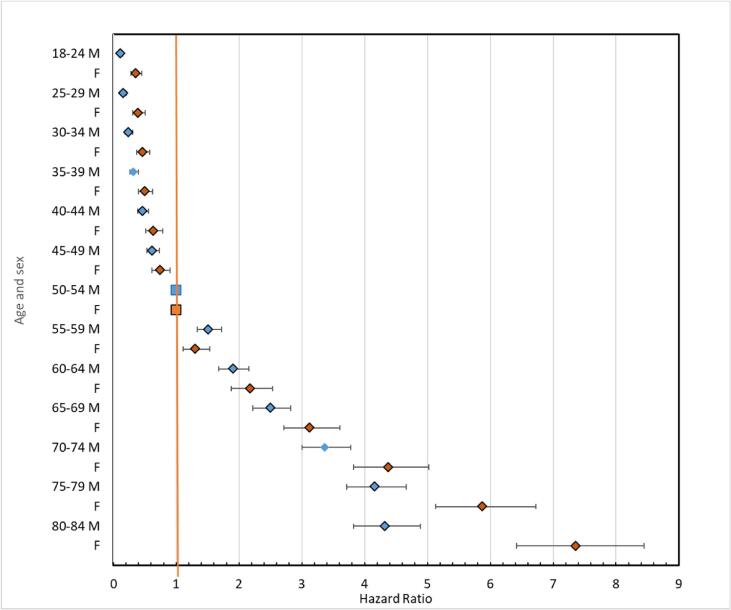
Hazard Ratios with 95 % confidence intervals for the outcome pulmonary emboli in 13 different age categories with 50–54 year of age as the reference for men (M; blue dots) and women (F; red dots). (For interpretation of the references to colour in this figure legend, the reader is referred to the web version of this article.)

Fig. 2 show HRs with 95 %-CIs for the outcome PE in thirteen different age categories with category 50–54 years of age as the reference for both men and women, respectively, revealing generally a higher age gradient for women than for men and importantly revealing an exponential increase in PE-risk with age for both sexes.

Table 2A-B present the numbers of vaccinated, person-years, events of pulmonary embolism and HRs for the outcome PE for the whole population age 18–84 years of age in the 28-day risk window for each of the combinations of vaccines in the primary vaccination schedule; dose I and II and following doses III – V of respective vaccine adjusted for sex and age (A) and for covariates included in the fully adjusted model (B). An increased risk was observed after vaccination with first dose of ChAdOx1 nCoV-19, an increase in risk of lower magnitude was observed after first dose of BNT162b2 but no increase in risk after mRNA1273. One single PE case was observed after the small number of NVX-CoV2373 doses administered (less than 10,000 not allowing for meaningful analyses). The high HRs observed for the combination of first dose BNT162b2 and second dose mRNA1273 is considered an anomaly as the number of PE cases were very few; n = 4.

**Table 2:** **A-B**. Hazard ratios, adjusted for sex and age and full model for the outcome pulmonary emboli in the 28 days window after primary vaccinations; dose I and II of and following doses III – V of respective vaccine (A) and for the covariates (B) included in the fully adjusted model.

Panel A Vaccination, dose number	N vaccinated	Person-years	Cases	HR adj sex & age	HR full adj
Reference: before first dose/ no vaccination	7,512,450	4,077,297	2,801	*Ref*	*ref*
1BNT	4,708,284	354,709	361	1.97 (1.75–2.21)	1.19 (1.06–1.34)
1BNT 2BNT	4,576,712	350,694	311	2.02 (1.78–2.30)	1.08 (0.95–1.22)
1BNT 2MOD	26,689	2,038	4	5.45 (2.04–14.56)	3.82 (1.43–10.19)
1BNT 2AZ	515	40	0		
1MOD	801,761	61,303	45	1.53 (1.14–2.06)	1.02 (0.76–1.37)
1MOD 2MOD	729,691	55,937	43	1.83 (1.35–2.49)	1.05 (0.78–1.43)
1MOD 2BNT	50,193	3,831	1	0.79 (0.11–5.60)	0.90 (0.13–6.39)
1MOD 2AZ	62	5	0		
1AZ	701,455	53,636	99	2.62 (2.13–3.22)	1.29 (1.05–1.59)
1AZ 2AZ	565,029	43,325	68	3.69 (2.87–4.74)	1.05 (0.81–1.35)
1AZ 2BNT	105,580	8,091	9	2.50 (1.30–4.84)	1.84 (0.96–3.56)
1AZ 2MOD	17,757	1,360	1	1.66 (0.23–11.77)	1.23 (0.17–8.77)
*Reference: dose 2*					
3BNT	3,154,403	239,249	302	3.83 (3.34–4.41)	1.13 (0.98–1.30)
3MOD	1,319,669	99,693	99	2.88 (2.33–3.57)	1.08 (0.87–1.33)
3AZ	69	5	0		
*Reference: dose 3*					
4BNT	1,666,429	123,926	163	4.38 (3.68–5.21)	0.97 (0.82–1.16)
4MOD	537,566	41,137	74	5.97 (4.68–7.61)	1.08 (0.85–1.38)
4AZ	4	0	0		
*Reference: dose 3*					
5BNT	1,005,999	75,105	137	6.09 (5.03–7.36)	0.96 (0.79–1.16)
5MOD	140,559	10,744	23	7.27 (4.77–11.07)	1.10 (0.72–1.68)
5AZ	8	1	0		
NOV (any dose number) *ref no vacc*	9,014	775	1	4.32 (0.61–30.70)	0.85 (0.12–6.05)
Outside the 28-days risk window, *ref no vacc*	6,176,335	7,448,000	7,914	3.21 (2.99–3.46)	1.13 (1.05–1.22)

Panel B Covariates	N vaccinated	Person-Years	Cases	HR _adj sex & age_	HR _full adj_
Age 2021				1.06 (1.06–1.06)	1.05 (1.05–1.06)
Male sex	37,78,719	66,19,069	6,415	*reference*	*reference*
Female sex	37,34,899	64,31,833	6,041	0.90 (0.87–0.93)	0.83 (0.80–0.86)
*Marital status*					
Married	30,55,967	53,16,198	6,061	*reference*	*reference*
Unmarried	32,52,080	55,66,299	2,785	1.16 (1.11–1.22)	1.12 (1.07–1.18)
Divorced	9,49,515	16,90,904	2,353	1.13 (1.08–1.18)	1.02 (0.97–1.07)
Widow/-er	2,56,056	4,77,501	1,257	1.12 (1.05–1.19)	1.04 (0.98–1.11)
*Country of birth*					
Sweden	60,71,525	1,06,41,409	10,255	*reference*	*reference*
Europe excl. Sweden	6,40,337	11,23,374	1,004	0.85 (0.80–0.91)	0.86 (0.81–0.92)
Outside Europe	8,01,756	12,86,119	1,197	1.56 (1.47–1.66)	1.36 (1.28–1.45)
Previous DVT	46,509	81,470	923	7.32 (6.85–7.83)	6.45 (6.02–6.92)
Antithrombotic treatment	2,90,423	5,32,034	963	0.75 (0.70–0.81)	0.49 (0.46–0.53)
In-hospital care	10,08,536	1,74,468	2,494	12.01 (11.48–12.56)	9.37 (8.93–9.82)
Number of drugs		n/a		1.06 (1.06–1.06)	1.03 (1.03–1.04)
Composite: diabetes or cardiovascular disease	7,85,260	14,20,371	2,645	0.96 (0.92–1.00)	0.67 (0.63–0.70)
Composite: obesity (diagnosis, surgery or drug treatment)	2,31,231	3,87,879	1,008	3.19 (2.99–3.40)	2.25 (2.10–2.41)
Composite: COPD, nicotine replacement therapy	1,82,018	3,21,963	1,390	2.68 (2.54–2.84)	1.66 (1.56–1.76)
Inflammatory bowel disease	87,041	1,50,316	294	1.93 (1.72–2.17)	1.37 (1.22–1.54)
Composite: autoimmune disease, diagnosis and treatment	31,194	54,145	106	1.72 (1.42–2.08)	1.21 (0.99–1.46)
Any malignancy	2,26,785	4,03,558	1,764	2.48 (2.35–2.61)	1.89 (1.79–1.99)
Severe kidney failure	7,941	12,310	34	1.61 (1.15–2.25)	0.46 (0.32–0.65)
Severe liver disease	7,270	12,528	35	1.65 (1.18–2.30)	0.76 (0.55–1.07)
Hormone treatment	7,92,502	8,50,437	921	1.13 (1.05–1.21)	1.05 (0.98–1.12)
Ciklosporin or tacrolimus	10,666	17,642	64	3.13 (2.45–4.01)	1.61 (1.25–2.07)
Thrombophilia	8,708	14,579	59	4.65 (3.60–6.01)	2.52 (1.94–3.27)

*Abbreviations*: BNT = BNT162b2, Comirnaty ® (Pfizer Biontech); MOD = mRNA-1273, Spikevax ® (Moderna); AZ = ChAdOx1 nCoV-19, Vaxzevria® (Astra-Zeneca); NUV = NVX-CoV2373, Nuvaxovid ® (Novavax). n/a depicts not applicable. Reference in panel B is the same as for analyses of dose 1 and 2 in panel A above.

Several covariates mediated increased risk for PE as for example age, with an increased risk for PE of 5 % per year with increasing age. Further, being unmarried, born outside Europe, experienced a previous DVT, having obesity, COPD, autoimmune diseases, any malignancy, organ transplant, thrombophilia or using oral contraceptive or sex-hormones implied higher risk.

**Fig. 3:**
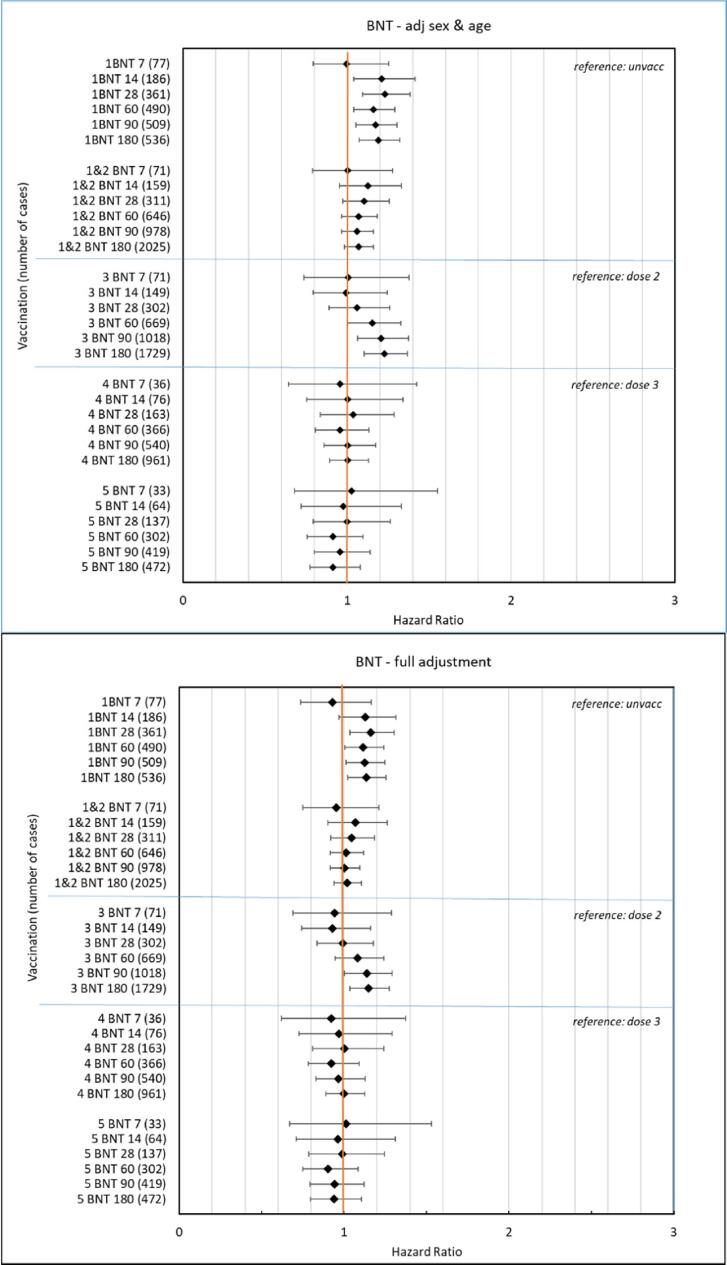
**A-B.** Forrest plot showing hazard ratios with 95 %-confidence intervals for the outcome pulmonary embolism at different risk windows up to 180 days, adjusted for sex and age in upper panel A and full adjustments in lower panel B for BNT 162b2 vaccine (BNT).

Fig. 3A-B show forest plots presenting HRs with 95 %-CIs for the outcome PE at different time windows of follow-up from 7 to 180 days after vaccination with BNT162b2, adjusted for age and sex in upper panel and adjusted, full model, in lower panel. Slightly increased risk for PE was observed after first dose of two in the primary vaccinations (point estimates; HRs around 1.15 in risk windows; 28 to 180 days) but not after the second dose. After the third dose a slightly increased risk was observed (point estimates; HRs around 1.15 in risk windows; 90 to 180 days). The overall pattern for booster doses four and five showed no increased risks.

**Fig. 4:**
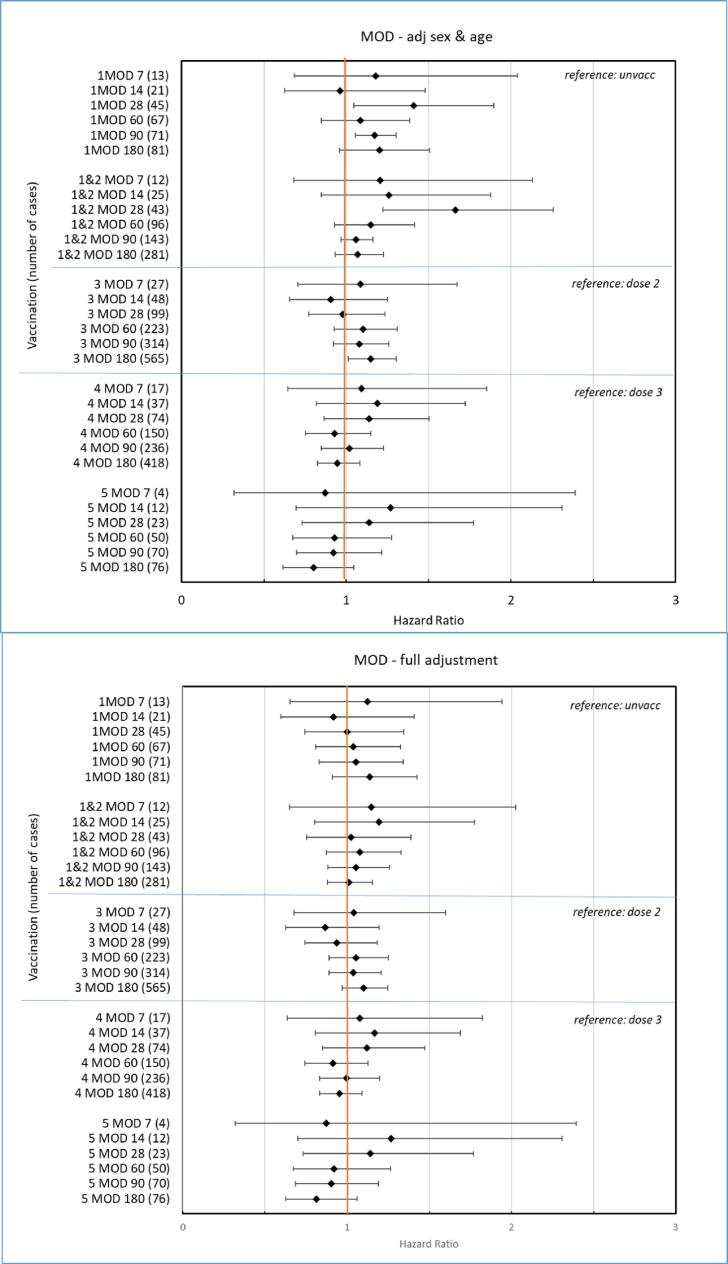
**A-B.** Forrest plot showing hazard ratios with 95 %-confidence intervals for the outcome pulmonary embolism at different risk windows up to 180 days, adjusted for sex and age in upper panel A and full adjustments in lower panel B for mRNA-1273 vaccine (MOD).

Fig. 4A–B present corresponding forest plots as in Fig. 3A–B after vaccinations with mRNA1273. No increased risks were observed in the fully adjusted models.

**Fig. 5:**
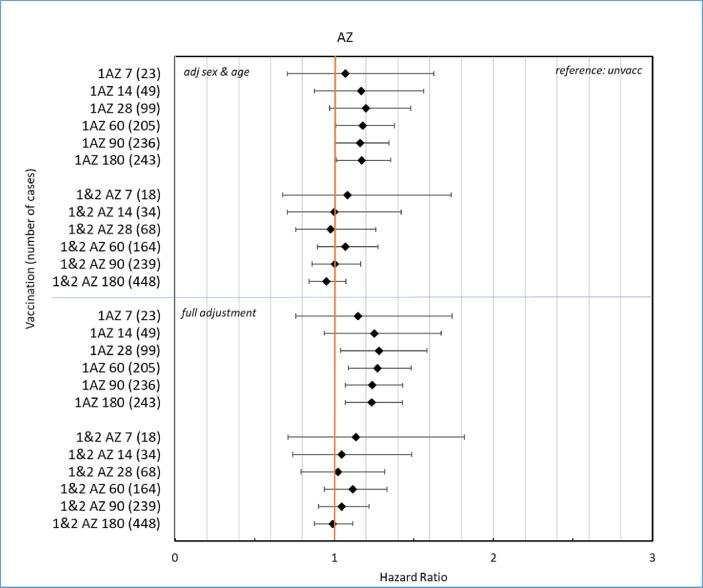
**A-B.** Forrest plot showing hazard ratios with 95 %-confidence intervals for the outcome pulmonary embolism at different risk windows up to 180 days, adjusted for sex and age in upper panel A and full adjustments in lower panel B for ChAdOx1 nCoV-19 vaccine (AZ).

Fig. 5A–B show corresponding forest plots as in Fig. 3A–B after vaccinations with ChAdOx1 nCoV-19. Increased risk for PE was observed after the first dose of ChAdOx1 nCoV-19 for all risk windows with HR ranging from 1.2 to 1.4 and lower CI close to 1.

Very few persons received more than two doses of ChAdOx1 nCoV-19, not allowing for further analyses.

In the appendix results corresponding to Table 2A–B are presented by age-groups 18–39 years (Table A1, A-B) 40–64 years (Table A2, A-B) and for 65–84 years (Table A3, A-B). In short, no increased risks were observed in the youngest age group, 18–39 years however the first doses of mRNA vaccines showed increased HRs the number of cases were few and 95 %-CIs were wide and included 1, thus not allowing any firm conclusions. However, in individuals aged 40–64 years the first dose of ChAdOx1 nCoV-19 in primary vaccinations was associated with increased PE-risk (HR, 1.91; 95 %-CI 1.01–3.59). In individuals aged 65–84 years, a slight increase in PE-risk was found after first and third dose of BNT162b2 but not for any dose of mRNA1273.

In Appendix Fig. A4 mean duration between doses one to five are presented and in Table S5 proportions of doses one to five in individuals aged 65–84 are shown where 94 % of the total amount of dose five was administered in this age group.

## Discussion

This nation-wide population-based study of more than 7.5 million individuals of which 80 % were vaccinated showed no strong associations between mRNA COVID-19 vaccination and risk of the serious medical emergency pulmonary embolism. Increased PE risk was observed for the first dose of ChAdOx1 nCoV-19 in the 28-to-180-day windows. For BNT162b2 an increased risk was observed for the first dose, however of a lower magnitude in the population aged 18–84 years but not for the second dose in the two-dose-primary-vaccination-scheme. Similarly, an increased risk was observed for the third BNT162b2 dose in the 90- and 180-days windows whereas no increase was observed for the fourth and fifth booster doses of BNT162b2. Subgroup analyses revealed that the slight increased PE risk for BNT162b2 was driven by the oldest age group, 65–84 years of age. For ChAdOx1 nCoV-19 a more pronounced PE risk was found in individuals in the age group 40–64 years of age.

There is a previous study in people aged 65 years and above during the first half of 2021 from the US on 28 days PE-risk with the two mRNA vaccines where BNT162b2, nor mRNA1273 were associated with increased PE-risk [11]. Another US-study in people 65 years and above showed inconsistent evidence under 28 days follow-up for PE after COVID-19 mRNA primary vaccinations or monovalent boosters [12]. Analyses of venous thromboembolic events (VTE) but not PE per se, in three Nordic countries; Finland, Norway and Denmark using national health registers, during the first half of 2021 including 5.3 million people vaccinated with either one or two doses, with 28 days follow-up, showed increased risk for VTE after ChAdOx1 nCoV-19 [17] which was more pronounced than slight increases observed after BNT162b2 and after mRNA1273. Sensitivity analyses were not consistent, and the authors called for confirmatory analyses. A study from Singapore during 2021 to September 2022 [10] with follow-up of 42 days after vaccinations with BNT162b2 or mRNA-1273, revealed no increased risk for PE. A study in people 75 years and above during December 2020 and April 2021 from France with short follow-up of 14 days after BNT162b2 vaccinations revealed no increased risk for PE [13] and a study from Israel on BNT162b2 up to 42 days after primary vaccinations did not either [14]. An increased risk of PE was seen after first dose of ChAdOx1 nCoV-19 and after BNT162b2 in a study from the UK using the Clinical Practise Research datalink [18]. During the vaccination campaign changes in viral biology occurred and mRNA-vaccines were updated with respect to new variants discovered [19] and a study from France showed no increased PE-risk for bivalent as compared to monovalent variants of the BNT162b2 vaccine [20].

The inconsistent risks observed in studies discussed above after vaccinations were generally of low magnitude. For comparison, the risk of PE is markedly increased in the acute phase of COVID-19-infection with a maximum around 70 days after a COVID-19 diagnosis and then for several months gradually fade with increasing time as observed in a recent register study with six months follow-up from Sweden [1] and are still increased up to 49 weeks after COVID-19 diagnosis as observed in a study from the UK [21]. In the present study, with longer follow-up after COVID-19 primary- and booster-vaccinations we did not observe increased risk for PE after mRNA vaccine doses four and five. Thus, according to our results repeated booster dosing seems safe from a usage perspective which may be of importance for possible future seasonal booster dosing against COVID-19.

The increased risk for PE in all time windows after ChAdOx1 nCoV-19 vaccination observed in the present study is in line with previous findings of thromboembolism associated with ChAdOx1 nCoV-19. A safety signal of thromboembolism after vaccinations with ChAdOx1 nCoV-19 was triggered in March 2021, then confirmed and documented as an ADR [22], [23], [24], [25], [18]. For ChAdOx1 nCoV-19 usage was restricted in Sweden in March 2021, not to be used in individuals younger than 65 years of age. Then, in the summer of 2021 national recommendations came on ending ChAdOx1 nCoV-19 usage, recommending mRNA vaccines to be used onwards and very few doses three to five for ChAdOx1 nCoV-19 were administered to less than 100 individuals. Later, a study from Norway and Denmark, two countries with similar health care systems as in Sweden, on the ChAdOx1 nCoV-19 vaccine revealed increased rates of thromboembolism within the first 28 days of primary vaccinations [26]. ChAdOx1 nCoV-19 was withdrawn from the market in April 2024.

Many risk factors have been identified in patients developing thromboembolism [27], [28]. Several of the risk factors and covariates adjusted for in the present study were observed to be associated with the outcome PE. Deep vein thrombosis, a predisposing thromboembolic factor was of great importance and further in-hospital care as a general marker of frailty also was. Further, obesity, COPD, any previous or current malignancy and thrombophilia were also associated with PE-risk. The increased risk of PE observed with the first dose of BNT162b2, driven by the higher age group may derive from selecting and prioritizing the frailest groups of people under high risk initially in the national vaccination campaigns phased plan, thus selection bias affecting our analyses cannot be ruled out. The third dose was also selected to be prioritized to those frailest under highest risk of complications to COVID-19, also in a phased manner before broader groups received their vaccine. Thus, a depletion of the most susceptible, may have occurred over time after initial vaccinations. Residual confounding as an explanation to the relatively low but still observed risks must be taken into consideration as important life-style factors such as obesity, smoking, excessive use of alcohol and a sedentary way of life are only partly and sometimes not at all identifiable in the registers used. Further, PE associated to pregnancy was not possible to analyse in this study since these specific diagnosis codes were not available.

The CoVacSafe-SE platform [9] for epidemiological surveillance to detect and characterise suspected adverse effects of COVID-19 vaccines in Sweden was set up following a Swedish government decision a governmental assignment to the SMPA on in depth safety follow-up of vaccines against COVID-19 (Dnr S2020/08531) [29].

The main strengths of the CoVacSafe-SE include the population-based cohort design with full nationwide coverage of the adult population and complete well-defined follow-up in a setting with national health services providing free access to healthcare. Other advantages include the availability and regular updates of complete data on COVID-19 vaccinations, and health outcomes and covariates from mandatory nationwide Swedish registers.

This study was limited by the fact that not all risk factors of importance for PE events were recorded in the national registers. This could therefore lead to unmeasured residual confounding of varying degrees. Further, there is no information on any data on pregnancies or conditions related to childbearing (i.e., chapter O in the ICD-coding system and not on Chapters P and Z). The associations observed for BNT126b2 first and third dose, with point estimates around 1.15, are admittedly weak. They are probably quite sensitive, i.e. also for low degrees of hypothetical correction of unmeasured residual confounding that would push the estimate towards the null.

COVID-19 infections are only captured if a laboratory test is performed. The availability of testing has varied over time and was commonly available from August 2020 to February 2022. The likelihood of being tested may depend on factors such as severity of symptoms, age, socioeconomic factors, and vaccination status. Hence, capture of mild disease is hampered by lacking obligate testing capacity during that period. Most important is the recommendation after February 2022 not to test when having mild symptoms. COVID-19 cases may thus go unrevealed and at least in some cases be related to PE among the vaccinated.

The definition of PE itself rely on contact with specialist outpatient care or hospitalisation for a registered diagnosis where a relatively large proportion (24 %) were handled in the policlinic, i.e. the out-patient setting. A possibility of differential misclassification of PE between vaccinated and unvaccinated may occur if the former is more prone to seek health care and thus have a greater chance to receive a PE diagnosis than the latter. In summary, residual confounding may occur even after diligent control for important covariates and risk factors.

## Conclusions

In this nation-wide study, no strong associations were found between repeated booster doses of COVID-19 vaccinations and pulmonary embolism. Small increases in risk for the first and third dose of BNT162b2 driven by those at higher age in the primary vaccination and the first of booster doses may be associated with prioritizing the most frailty groups of people in the initial parts of the phased vaccination campaign, thus a selection bias and depletion by susceptibility may not have been fully ruled out. Residual unmeasured confounding is important to consider as it may occur even after diligent control for registered covariates. Increased PE-risk observed after ChAdOx1 nCoV-19 is in line with the previously established causal relationship between the adenovirus vector vaccine ChAdOx1 nCoV-19 and venous thromboembolism.

## CRediT authorship contribution statement

**Björn Zethelius:** Writing – review & editing, Writing – original draft, Visualization, Project administration, Conceptualization. **Sofia Attelind:** Writing – review & editing, Conceptualization. **Gabriel Westman:** Writing – review & editing, Conceptualization. **Rickard Ljung:** Writing – review & editing, Supervision, Conceptualization. **Anders Sundström:** Writing – review & editing, Visualization, Formal analysis, Data curation, Conceptualization.

## Funding

This research was performed at the SMPA which is a governmental regulatory agency. No external funding was received.

## Declaration of competing interest

The authors declare that they have no known competing financial interests or personal relationships that could have appeared to influence the work reported in this paper.

## Data Availability

Data will be made available on request.
