# Supplementary material for: Pulmonary embolism after SARS-CoV-2 vaccination
Source: Vaccine X. 2024 Oct 15;21:100571. doi: 10.1016/j.jvacx.2024.100571 (PMC11513630; doi:10.1016/j.jvacx.2024.100571)
Supplement: Supplementary Data 1 [file mmc1.docx]

**APPENDIX**

# Pulmonary embolism after vaccination against SARS-CoV-2

# Report from the COvid-19 VACcination register SAFEty study in Sweden

**Zethelius B. et al**

**Tables A1-A5 and figure A1**

**Appendix, table A1 A - B – Age 18-39.** Hazard ratios, for the outcome pulmonalis emboli up to 28 days after primary vaccinations; dose I and II and following doses III – V of respective vaccine adjusted for sex and age (A) and for all covariates (B) included in the fully adjusted model in the subgroup 18-39 years old.

| **Panel A**  **Vaccination, dose number** | | **Individuals** | **Person-Years** | **Cases** | **HR _adj sex & age_** | **HR _full adj_** |
| --- | --- | --- | --- | --- | --- | --- |
| *Reference: before first dose/no vaccination* | | 2,593,736 | 1,830,927 | 298 | *ref* | *ref* |
| 1BNT | 1,525,594 | | 115,627 | 28 | 1.62 (1.06-2.49) | 1.49 (0.98-2.27) |
| 1BNT 2BNT | | 1,451,164 | 111,133 | 16 | 0.84 (0.49-1.44) | 0.81 (0.47-1.39) |
| 1BNT 2MOD | | 14,913 | 1,140 | 0 |  |  |
| 1BNT 2AZ | | 29 | 2 | 0 |  |  |
| 1MOD | | 352,609 | 26,962 | 8 | 2.02 (0.98-4.18) | 1.89 (0.92-3.88) |
| 1MOD 2MOD | | 298,524 | 22,883 | 5 | 1.33 (0.53-3.30) | 1.29 (0.52-3.20) |
| 1MOD 2BNT | | 41,075 | 3,137 | 1 | 2.40 (0.33-17.26) | 2.17 (0.30-15.61) |
| 1MOD 2AZ | | 7 | 1 | 0 |  |  |
| 1AZ | | 72,528 | 5,532 | 1 | 0.96 (0.13-6.93) | 0.97 (0.13-7.05) |
| 1AZ 2AZ | | 26,489 | 2,029 | 0 |  |  |
| 1AZ 2BNT | | 36,386 | 2,788 | 0 |  |  |
| 1AZ 2MOD | | 6,252 | 479 | 0 |  |  |
| *Reference: dose 2* | |  |  |  |  |  |
| 3BNT | | 741,379 | 55,950 | 12 | 1.45 (0.65-3.25) | 1.23 (0.56-2.73) |
| 3MOD | | 219,381 | 16,480 | 3 | 0.87 (0.24-3.12) | 0.75 (0.21-2.70) |
| 3AZ | | 4 | 0 | 0 |  |  |
| *Reference: dose 3* | |  |  |  |  |  |
| 4BNT | | 127,365 | 670 | 1 | 0.65 (0.08-5.37) | 0.47 (0.06-3.85) |
| 4MOD | | 8,863 | 0 | 1 | 7.29 (0.88-60.53) | 4.86 (0.59-40.21) |
| 4AZ | | 0 | 9,010 | 0 |  |  |
| *Reference: dose 3* | |  |  |  |  |  |
| 5BNT | | 3,864 | 278 | 0 |  |  |
| 5MOD | | 225 | 17 | 0 |  |  |
| 5AZ | | 0 | 0 | 0 |  |  |
| NOV (any dose number) *ref no vacc* | | 736 | 68 | 0 |  |  |
| Outside the 28-days  risk window, *ref no vacc* | | 1,939,183 | 2,108,026 | 361 | 1.08 (0.87-1.35) | 0.96 (0.77-1.20) |

**Appendix, table A1 A - B – Age 18-39 continued**

|  |  |  |  |  |  |
| --- | --- | --- | --- | --- | --- |
| **Panel B**  **Covariates** *ref no vacc* | **Individuals** | **Person-years** | **Cases** | **HR _adj sex & age_** | **HR _full adj_** |
| Age2021 |  |  |  | 1.05 (1.03-1.06) | 1.05 (1.03-1.06) |
| Male sex | 1,335,959 | 2,267,289 | 324 | *Ref* | *Ref* |
| Female sex | 1,257,852 | 2,045,853 | 411 | 1.40 (1.21-1.62) | 0.71 (0.60-0.84) |
| *Marital status* |  |  |  |  |  |
| Married | 462,950 | 743,568 | 136 | *Ref* | *Ref* |
| Unmarried | 2,053,496 | 3,442,069 | 561 | 1.24 (1.01-1.52) | 1.15 (0.93-1.41) |
| Divorced | 76,044 | 125,301 | 36 | 1.51 (1.04-2.17) | 1.31 (0.90-1.89) |
| Widow/-er | 1,321 | 2,203 | 2 | 4.40 (1.09-17.76) | 4.75 (1.18-19.22) |
| *Country of birth* |  |  |  |  |  |
| Sweden | 2,116,144 | 3,525,922 | 623 | *Ref* | *Ref* |
| Europe excl Sweden | 157,499 | 261,947 | 36 | 0.68 (0.49-0.96) | 0.77 (0.55-1.09) |
| Outside Europe | 320,168 | 525,274 | 76 | 0.76 (0.60-0.96) | 0.83 (0.65-1.07) |
| Previous DVT | 3,928 | 6,312 | 51 | 44.1 (33.1-58.7) | 18.2 (12.6-26.1) |
| Antithrombotic treatment | 5,375 | 8,992 | 24 | 14.6 (9.73-22.00) | 1.40 (0.84-2.31) |
| In-hospital care | 272,560 | 41,396 | 111 | 16.9 (13.7-20.7) | 11.6 (9.28-14.5) |
| Number of drugs |  |  |  | 1.12 (1.11-1.13) | 1.05 (1.03-1.06) |
| Composite: diabetes or cardiovascular disease | 43,814 | 73,228 | 28 | 2.17 (1.49-3.17) | 0.96 (0.65-1.43) |
| Composite: obesity (diagnosis, surgery or drug treatment) | 88,065 | 141,873 | 114 | 4.79 (3.89-5.89) | 3.75 (3.01-4.68) |
| Composite: COPD, nicotine replacement therapy | 12,380 | 20,538 | 15 | 3.63 (2.18-6.07) | 1.83 (1.08-3.09) |
| Inflammatory bowel disease | 26,477 | 43,508 | 21 | 2.76 (1.79-4.26) | 1.69 (1.08-2.63) |
| Composite: autoimmune disease, diagnosis and treatment | 5,168 | 8,404 | 4 | 2.49 (0.93-6.66) | 1.26 (0.47-3.39) |
| Any malignancy | 8,288 | 13,551 | 24 | 9.15 (6.08-13.77) | 4.36 (2.85-6.67) |
| Severe kidney failure | 678 | 1,069 | 1 | 5.20 (0.73-36.89) | 0.75 (0.10-5.89) |
| Severe liver disease | 304 | 520 | 0 |  |  |
| Hormone treatment | 299,493 | 305,817 | 155 | 4.07 (3.33-4.97) | 4.50 (3.68-5.50) |
| Ciklosporin or tacrolimus | 1,698 | 2,770 | 3 | 6.09 (1.96-18.90) | 1.59 (0.48-5.31) |
| Thrombophilia | 3,495 | 5,564 | 12 | 9.92 (5.59-17.61) | 2.93 (1.57-5.47) |

Abbrevations: BNT = BNT162b2, Comirnaty ® (Pfizer Biontech); MOD = mRNA-1273, Spikevax ® (Moderna); AZ = ChAdOx1 nCoV-19, Vaxzevria® (Astra-Zeneca); NUV= NVX-CoV2373, Nuvaxovid ® (Novavax). Reference in panel B is the same as for analyses of dose 1 and 2 in panel A above.

**Appendix, table A2 A - B – Age 40-64.** Hazard ratios, for the outcome pulmonalis emboli up to 28 days after primary vaccinations; dose I and II and following doses III – V of respective vaccine adjusted for sex and age (A) and for all covariates (B) included in the fully adjusted model in the subgroup 40-64 years old.

| **Panel A**  **Vaccination, dose number** | **Individuals** |  | **Cases** | **HR _adj sex & age_** | **HR _full adj_** |
| --- | --- | --- | --- | --- | --- |
| *Reference: Before first dose/no vaccination* | 3,059,887 | 1,595,115 | 1,008 | *ref* | *ref* |
| 1BNT | 2,103,964 | 159,368 | 100 | 1.00 (0.79-1.26) | 0.99 (0.79-1.24) |
| 1BNT 2BNT | 2,061,832 | 158,049 | 99 | 0.99 (0.79-1.26) | 0.96 (0.76-1.22) |
| 1BNT 2MOD | 9,839 | 750 | 2 | 4.70 (1.17-18.91) | 4.12 (1.03-16.58) |
| 1BNT 2AZ | 132 | 10 | 0 |  |  |
| 1MOD | 307,877 | 23,539 | 13 | 0.99 (0.57-1.72) | 0.92 (0.53-1.60) |
| 1MOD 2MOD | 293,541 | 22,505 | 16 | 1.25 (0.75-2.07) | 1.16 (0.70-1.92) |
| 1MOD 2BNT | 7,314 | 557 | 0 |  |  |
| 1MOD 2AZ | 23 | 2 | 0 |  |  |
| 1AZ | 121,315 | 9,250 | 10 | 1.83 (0.97-3.45) | 1.91 (1.01-3.59) |
| 1AZ 2AZ | 49,919 | 3,824 | 3 | 1.38 (0.44-4.31) | 1.41 (0.45-4.43) |
| 1AZ 2BNT | 57,155 | 4,380 | 7 | 2.94 (1.38-6.27) | 2.93 (1.38-6.24) |
| 1AZ 2MOD | 9,386 | 719 | 0 |  |  |
| *Reference: dose 2* |  |  |  |  |  |
| 3BNT | 1,143,729 | 86,135 | 59 | 0.89 (0.62-1.29) | 0.81 (0.56-1.16) |
| 3MOD | 752,716 | 56,606 | 37 | 0.92 (0.61-1.39) | 0.89 (0.59-1.34) |
| 3AZ | 17 | 1 | 0 |  |  |
| *Reference: dose 3* |  |  |  |  |  |
| 4BNT | 591,764 | 42,758 | 31 | 1.33 (0.83-2.12) | 1.08 (0.68-1.72) |
| 4MOD | 95,295 | 7,255 | 5 | 1.20 (0.47-3.04) | 0.93 (0.37-2.34) |
| 4AZ | 0 | 0 | 0 |  |  |
| *Reference: dose 3* |  |  |  |  |  |
| 5BNT | 57,534 | 4,131 | 5 | 1.64 (0.64-4.16) | 1.08 (0.43-2.75) |
| 5MOD | 6,990 | 530 | 0 |  |  |
| 5AZ | 2 | 0 | 0 |  |  |
| NOV (any dose number) *ref no vacc* | 2,313 | 200 | 1 | 7.58 (1.06-54.02) | 4.38 (0.61-31.33) |
| Outside the 28-days  risk window, *ref no vacc* | 2,519,876 | 3,052,477 | 2,076 | 1.09 (0.97-1.24) | 1.01 (0.90-1.14) |

**Appendix, table A2 A-B – Age 40-64 continued**

|  |  |  |  |  |  |
| --- | --- | --- | --- | --- | --- |
| **Panel B**  **Covariates** *ref no vacc* | **Individuals** | **Person-Years** | **Cases** | **HR _adj sex & age_** | **HR _full adj_** |
| Age2021 |  |  |  | 1.07 (1.07-1.08) | 1.06 (1.05-1.06) |
| Male sex | 1,544,716 | 2,667,622 | 2,050 | *Ref* | *Ref* |
| Female sex | 1,515,429 | 2,560,539 | 1,422 | 0.71 (0.67-0.76) | 0.65 (0.60-0.69) |
| *Marital status* |  |  |  |  |  |
| Married | 1,567,955 | 2,633,768 | 1,586 | *Ref* | *Ref* |
| Unmarried | 948,430 | 1,651,613 | 1,168 | 1.26 (1.17-1.36) | 1.14 (1.05-1.23) |
| Divorced | 510,746 | 884,669 | 669 | 1.18 (1.08-1.29) | 1.03 (0.94-1.13) |
| Widow/-er | 33,014 | 58,111 | 49 | 1.14 (0.86-1.52) | 1.05 (0.79-1.39) |
| *Country of birth* |  |  |  |  |  |
| Sweden | 2,388,373 | 4,100,379 | 2,838 | *Ref* | *Ref* |
| Europe excl Sweden | 300,458 | 513,511 | 308 | 0.88 (0.78-0.99) | 0.90 (0.80-1.01) |
| Outside Europe | 371,314 | 614,271 | 326 | 0.88 (0.78-0.98) | 0.85 (0.76-0.96) |
| Previous DVT | 17,886 | 30,086 | 310 | 14.49 (12.9-16.3) | 9.92 (8.72-11.3) |
| Antithrombotic treatment | 55,032 | 96,178 | 191 | 2.27 (1.95-2.62) | 0.76 (0.64-0.89) |
| In-hospital care | 276,820 | 47,100 | 651 | 22.7 (20.8-24.7) | 14.3 (13.1-15.8) |
| Number of drugs |  |  |  | 1.09 (1.08-1.09) | 1.05 (1.04-1.05) |
| Composite: diabetes or cardiovascular disease | 235,569 | 410,573 | 460 | 1.35 (1.22-1.49) | 0.68 (0.61-0.76) |
| Composite: obesity (diagnosis, surgery or drug treatment) | 92,330 | 154,065 | 381 | 4.22 (3.79-4.69) | 2.38 (2.12-2.67) |
| Composite: COPD, nicotine replacement therapy | 60,921 | 106,656 | 216 | 2.74 (2.39-3.15) | 1.32 (1.14-1.52) |
| Inflammatory bowel disease | 36,034 | 61,097 | 92 | 2.28 (1.86-2.81) | 1.35 (1.09-1.66) |
| Composite: autoimmune disease, diagnosis and treatment | 15,747 | 26,604 | 36 | 2.01 (1.45-2.79) | 1.23 (0.88-1.71) |
| Any malignancy | 63,916 | 108,774 | 341 | 4.21 (3.76-4.71) | 2.59 (2.30-2.91) |
| Severe kidney failure | 2,846 | 4,437 | 13 | 3.66 (2.12-6.30) | 0.60 (0.34-1.07) |
| Severe liver disease | 2,668 | 4,472 | 8 | 2.10 (1.05-4.21) | 0.62 (0.31-1.24) |
| Hormone treatment | 271,700 | 283,047 | 209 | 1.19 (1.03-1.37) | 0.99 (0.85-1.14) |
| Ciklosporin or tacrolimus | 5,274 | 8,587 | 25 | 3.78 (2.55-5.61) | 1.22 (0.80-1.85) |
| Thrombophilia | 3,368 | 5,581 | 24 | 7.23 (4.84-10.80) | 1.62 (1.07-2.45) |

Abbrevations: BNT = BNT162b2, Comirnaty ® (Pfizer Biontech); MOD = mRNA-1273, Spikevax ® (Moderna); AZ = ChAdOx1 nCoV-19, Vaxzevria® (Astra-Zeneca); NUV= NVX-CoV2373, Nuvaxovid ® (Novavax). Reference in panel B is the same as for analyses of dose 1 and 2 in panel A above.

**Appendix, table A3 A-B – Age 65-84**

**Appendix, table A3 A - B – Age 65-84.** Hazard ratios, for the outcome pulmonalis emboli up to 28 days after primary vaccinations; dose I and II and following doses III – V of respective vaccine adjusted for sex and age (A) and for all covariates (B) included in the fully adjusted model in the subgroup 65-84 years old.

| **Panel A**  **Vaccination, dose number** | **Individuals** | **Peron-Years** | **Cases** | **HR _adj sex & age_** | **HR _full adj_** |
| --- | --- | --- | --- | --- | --- |
| *Reference: before first dose/no vaccination* | 1,858,827 | 651,254 | 1495 | *Ref* | *ref* |
| 1BNT | 1,078,726 | 79,714 | 233 | 1.32 (1.13-1.54) | 1.24 (1.07-1.44) |
| 1BNT 2BNT | 1,063,716 | 81,512 | 196 | 1.24 (1.05-1.47) | 1.17 (0.99-1.38) |
| 1BNT 2MOD | 1,937 | 148 | 2 | 7.06 (1.76-28.33) | 5.45 (1.36-21.87) |
| 1BNT 2AZ | 354 | 27 | 0 |  |  |
| 1MOD | 141,275 | 10,802 | 24 | 0.93 (0.62-1.40) | 0.92 (0.61-1.38) |
| 1MOD 2MOD | 137,626 | 10,550 | 22 | 1.01 (0.65-1.54) | 0.96 (0.63-1.48) |
| 1MOD 2BNT | 1,804 | 137 | 0 |  |  |
| 1MOD 2AZ | 32 | 2 | 0 |  |  |
| 1AZ | 507,612 | 38,854 | 88 | 1.17 (0.92-1.48) | 1.24 (0.98-1.57) |
| 1AZ 2AZ | 488,621 | 37,472 | 65 | 1.06 (0.80-1.39) | 1.10 (0.84-1.45) |
| 1AZ 2BNT | 12,039 | 922 | 2 | 1.34 (0.33-5.36) | 1.04 (0.26-4.16) |
| 1AZ 2MOD | 2,119 | 162 | 1 | 3.77 (0.53-26.83) | 2.91 (0.41-20.74) |
| *Reference: dose 2* |  |  |  |  |  |
| 3BNT | 1,269,295 | 97,164 | 231 | 1.29 (1.08-1.55) | 1.26 (1.05-1.51) |
| 3MOD | 347,572 | 26,608 | 59 | 1.21 (0.91-1.61) | 1.18 (0.89-1.57) |
| 3AZ | 48 | 4 | 0 |  |  |
| *Reference: dose 3* |  |  |  |  |  |
| 4BNT | 947,300 | 72,158 | 131 | 0.94 (0.72-1.22) | 0.94 (0.72-1.21) |
| 4MOD | 433,408 | 33,212 | 68 | 1.04 (0.76-1.42) | 1.05 (0.78-1.43) |
| 4AZ | 4 | 0 | 0 |  |  |
| *Reference: dose 3* |  |  |  |  |  |
| 5BNT | 944,601 | 70,696 | 132 | 0.94 (0.72-1.23) | 0.96 (0.74-1.24) |
| 5MOD | 133,344 | 10,197 | 23 | 1.12 (0.71-1.77) | 1.15 (0.73-1.82) |
| 5AZ | 6 | 0 | 0 |  |  |
| NOV (any dose number) *ref no vacc* | 5,965 | 506 | 0 |  |  |
| Outside the 28-days  risk window, *ref no vacc* | 1,717,276 | 2,287,498 | 5,477 | 1.33 (1.19-1.48) | 1.24 (1.12-1.38) |

**Appendix, table A3 A-B – Age 65-84 continued**

|  |  |  |  |  |  |
| --- | --- | --- | --- | --- | --- |
| **Panel B**  **Covariates** *ref no vacc* | **Individuals** | **Person-Years** | **Cases** | **HR _adj sex & age_** | **HR _full adj_** |
| Age2021 |  |  |  | 1.05 (1.05-1.05) | 1.04 (1.04-1.05) |
| Male sex | 898,044 | 1,684,159 | 4,041 | *Ref* | *Ref* |
| Female sex | 961,618 | 1,825,441 | 4,208 | 0.95 (0.91-0.99) | 0.93 (0.89-0.97) |
| *Marital status* |  |  |  |  |  |
| Married | 1,025,062 | 1,938,862 | 4,339 | *Ref* | *Ref* |
| Unmarried | 250,154 | 472,616 | 1,056 | 1.08 (1.01-1.16) | 1.05 (0.98-1.12) |
| Divorced | 362,725 | 680,934 | 1,648 | 1.10 (1.03-1.16) | 0.98 (0.93-1.04) |
| Widow/-er | 221,721 | 417,187 | 1,206 | 1.12 (1.05-1.20) | 1.05 (0.98-1.12) |
| *Country of birth* |  |  |  |  |  |
| Sweden | 1,567,008 | 3,015,109 | 6,794 | *Ref* | *Ref* |
| Europe excl Sweden | 182,380 | 347,916 | 660 | 0.85 (0.78-0.92) | 0.85 (0.79-0.92) |
| Outside Europe | 110,274 | 146,574 | 795 | 2.58 (2.39-2.77) | 2.04 (1.89-2.20) |
| Previous DVT | 24,695 | 45,071 | 562 | 5.36 (4.92-5.84) | 5.05 (4.62-5.51) |
| Antithrombotic treatment | 230,016 | 426,864 | 748 | 0.62 (0.58-0.67) | 0.47 (0.43-0.51) |
| In-hospital care | 459,156 | 85,971 | 1,732 | 9.94 (9.42-10.48) | 7.99 (7.55-8.45) |
| Number of drugs |  |  |  | 1.05 (1.04-1.05) | 1.02 (1.02-1.03) |
| Composite: diabetes or cardiovascular disease | 505,877 | 936,569 | 2,157 | 0.88 (0.84-0.93) | 0.69 (0.65-0.73) |
| Composite: obesity (diagnosis, surgery or drug treatment) | 50,836 | 91,941 | 513 | 2.54 (2.32-2.78) | 1.89 (1.72-2.08) |
| Composite: COPD, nicotine replacement therapy | 108,717 | 194,769 | 1,159 | 2.67 (2.50-2.84) | 1.81 (1.70-1.94) |
| Inflammatory bowel disease | 24,530 | 45,712 | 181 | 1.73 (1.49-2.00) | 1.31 (1.13-1.51) |
| Composite: autoimmune disease, diagnosis and treatment | 10,279 | 19,136 | 66 | 1.56 (1.22-1.98) | 1.18 (0.92-1.50) |
| Any malignancy | 154,581 | 281,233 | 1,399 | 2.22 (2.09-2.35) | 1.77 (1.67-1.88) |
| Severe kidney failure | 4,417 | 6,804 | 20 | 1.16 (0.75-1.80) | 0.40 (0.26-0.62) |
| Severe liver disease | 4,298 | 7,536 | 27 | 1.54 (1.06-2.25) | 0.80 (0.55-1.17) |
| Hormone treatment | 221,309 | 261,572 | 557 | 0.91 (0.83-0.99) | 0.87 (0.80-0.96) |
| Ciklosporin or tacrolimus | 3,694 | 6,284 | 36 | 2.63 (1.90-3.65) | 1.58 (1.13-2.21) |
| Thrombophilia | 1,845 | 3,434 | 23 | 2.81 (1.86-4.22) | 2.00 (1.32-3.02) |

Abbrevations: BNT = BNT162b2, Comirnaty ® (Pfizer Biontech); MOD = mRNA-1273, Spikevax ® (Moderna); AZ = ChAdOx1 nCoV-19, Vaxzevria® (Astra-Zeneca); NUV= NVX-CoV2373, Nuvaxovid ® (Novavax). Reference in panel B is the same as for analyses of dose 1 and 2 in panel A above.

**Appendix, Table A4.** Listing of variable definitions used where data from compulsory registers on individuals, their age, marital status, and country of birth held by Statistics Sweden and data on medicines used and on diseases held by the National Board of Health and Welfare.

Number of drugs used: Number of substances (ATC-code, 7 positions) dispensed for 12 months preceding study start.

2 Combined hormonal contraceptives: ATC-codes; G03AA (Progestogens and estrogens, fixed combinations), G03AB (Progestogens and estrogens, sequential preparations) dispensed during follow-up.

3 Hormones: ATC-codes; G03C (Estrogens), G03DB08 (Dienogest), G03DC02 (Norethisterone), G03F (Progestogens and estrogens in combination) dispensed during follow-up.

4 Various hormones: ATC-codes; G03GA (Gonadotropins), G03HB01 (Cyproterone and estrogen), G03XC01 (Raloxifene), G03XX01 (Prasterone) dispensed during follow-up.

5 Immunosuppressives, ciclosporin and tacrolimus used by transplanted patients: Dispensed for 12 months preceding study start.

^6^ Previous deep vein thrombosis (DVT): ICD codes 80.1, 80.2 or 80.3 between 2015 and study start.

7 Anti coagulants: ATC-codes; B01AA (Vitamin K antagonists), B01AE (Direct thrombin inhibitors), B01AF (Direct factor Xa inhibitors) dispensed for 12 months preceding study start.

^8^ Nicotine replacement therapy: ATC code N07BA dispensed for 12 months preceding study start.

9 Chronic Obstructive Pulmonary Disease (COPD): ICD code J44 between 2017 and study start.

1^0^ Diabetes: ICD codes; E10, E11, E12, E13, E14 between 2017 and study start.

1^1^ Antidiabetic drugs used: ATC codes A10 dispensed for 12 months preceding study start.

^12^ Cardio-vascular diseases (CVDs): ICD codes; I05-I09, I11, I20-I28, I34-I37, I39, I42, I43, I46, I48-I50 between 2017 and study start.

1^3^ Obesity registry diagnosis: ICD code E66 between 2015 and study start.

1^4^ Obesity surgery between 2015 and study start.

^15^ Anti-obesity drugs: ATC codes A08 dispensed between 2015 and study start.

^16^ Autoimmune diseases: ICD-codes; D86, G35, K50, K51, L40, M05-M09, M13, M14, M45 between 2017 and study start.

^17^ Drugs against autoimmune diseases: ATC codes; L04AB, L04AC, L04AA29, L04AA37, L04AA44 dispensed for 12 months preceding study start.

The composite of autoimmune was a diagnosis registered two or more times and two or more dispensations of drugs.

^18^ Inflammatory Bowel Disease (IBD): ICD codes; K50, K51 between 2015 and study start.

^19^ Cancer: Any malignancy in the cancer register between 2015 and study start.

^20^ Sever kidney disease: ICD code N18.5 (chronic kidney disease, stage 5).

^21^ Sever hepatic disease: ICD-code K74 (fibrosis and cirrhosis of liver).

^22^ Thrombophilia: ICD-codes; D685A (Activated protein C resistance [factor V Leiden mutation]). D685E (Prothrombin gene mutation) between 2017 and start of study.

^23^ Pulmonary emboli: ICD-code I26.

**Appendix, Figure A1.** Time span between respective COVID-19-vaccine doses and proportion of doses in the subgroup aged 65-84 years of age (grey line).


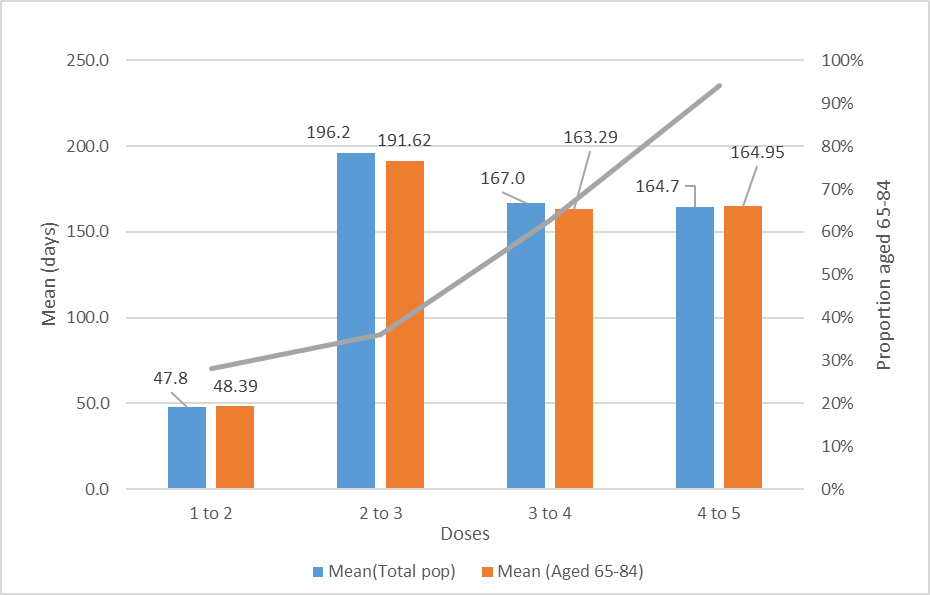


**Appendix, Table A5.** Number of COVID-19-vaccine doses given and number of days between respective doses and proportion aged 65-84 years of age who received two, three, four or five vaccine doses.

|  | Total population | | Subgroup 65-84 years of age | |  |  |
| --- | --- | --- | --- | --- | --- | --- |
| Dose-interval | Doses (n) | Dose interval (days) | Doses (n) | % of total | Dose interval (days) |  |
| **1 to 2** | 6,072,228 | 48 | 1,708,248 | 28.1% | 48 |  |
| **2 to 3** | 4,474,141 | 196 | 1,616,916 | 36.1% | 192 |  |
| **3 to 4** | 2,203,999 | 167 | 1,380,697 | 62.6% | 163 |  |
| **4 to 5** | 1,146,566 | 165 | 1,078,325 | 94.0% | 165 |  |
